# Integrated Multi‐Omics and Single‐Cell Analyses Implicate NOX4 as an Icariin‐Associated Oxidative Stress Regulatory Node in Gastric Cancer

**DOI:** 10.1002/fsn3.72390

**Published:** 2026-09-27

**Authors:** Na Liang, Junyan Li, Zuchao Luo, Zijuan Song, Liyuan Zhang, Chengyi Zou, Chunming Li, Bochen Pan, Jiawei Yang

**Affiliations:** ^1^ Department of Biochemistry, School of Preclinical Medicine Zunyi Medical University Zunyi Guizhou China; ^2^ Department of Pathology Zunyi Medical University Zunyi Guizhou China; ^3^ Department of Medical Genetics Zunyi Medical University Zunyi China; ^4^ Guizhou Biomanufacturing Laboratory Affiliated Hospital of Zunyi Medical University Zunyi China; ^5^ Key Laboratory of Cell Engineering of Guizhou Province Affiliated Hospital of Zunyi Medical University Zunyi China; ^6^ Department of Urology, School of Medicine, Shanghai Tenth People's Hospital Tongji University Shanghai China

**Keywords:** dietary polyphenols, gastric cancer, icariin, non‐communicable chronic diseases, NOX4

## Abstract

Icariin, a prenylated flavonoid glycoside from Epimedium species, is a representative dietary polyphenol with antioxidant, anti‐inflammatory, and antitumor properties. Gastric cancer (GC) is a major non‐communicable chronic disease characterized by complex molecular alterations and dysregulated redox homeostasis, yet the molecular targets underlying the potential anti‐GC activity of icariin remain poorly defined. In this study, we integrated network pharmacology, bulk transcriptomic analysis (TCGA‐STAD; GSE15497, GSE84437, GSE84433, and GSE26253), and single‐cell RNA sequencing (GSE183904; 142,053 cells) to identify candidate icariin‐associated genes in GC. NOX4 expression, prognostic value, and tumor microenvironment characteristics were systematically evaluated. A NOX4‐positive endothelial cell (NOX4pos_Endo)‐associated molecular subtyping strategy and a LASSO‐Cox prognostic model were constructed and validated. Molecular docking and molecular dynamics simulation were performed to explore a potential interaction between icariin and NOX4, and AGS cells were used for in vitro validation. Network pharmacology identified 24 overlapping icariin–GC targets, among which NOX4 showed prominent prognostic relevance. NOX4 was upregulated in GC and associated with unfavorable prognosis across multiple cohorts. At the single‐cell level, NOX4 was predominantly expressed in an endothelial subpopulation characterized by angiogenesis‐, adhesion‐, and extracellular matrix remodeling‐related transcriptional features. Consensus clustering identified two molecular subtypes with distinct clinical outcomes and biological characteristics. A 5‐gene prognostic model comprising SPIRE1, PLCH1, KCNS3, SLC27A2, and GRP showed moderate predictive performance across independent cohorts. Molecular docking suggested a potential icariin–NOX4 interaction involving Ser576 and Trp377, while molecular dynamics simulation supported the stability of the predicted binding pose. In AGS cells, icariin treatment was associated with reduced NOX4 expression and intracellular ROS levels. Overall, these findings identify NOX4 as a candidate icariin‐associated regulatory node in GC and suggest that NOX4/ROS‐related signaling may contribute to the cellular response to icariin. Further experimental studies are required to determine whether NOX4 is a direct molecular target of icariin and to clarify its therapeutic relevance.

## Graphical abstract

NOX4 represents a candidate icariin‐associated regulatory node in gastric cancer and may contribute to the cellular response to icariin through redox‐related signaling.

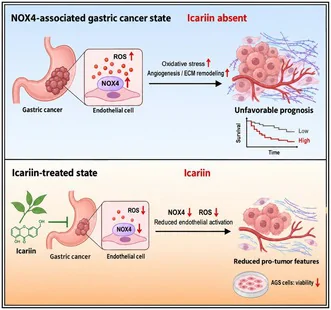

## 1 Introduction

Dietary polyphenols, the most abundant secondary metabolites in plant‐based foods, have attracted increasing attention for their preventive potential against NCCDs, including malignant tumors, through their antioxidant and anti‐inflammatory activities. Among these, icariin—a prenylated flavonoid glycoside isolated from Epimedium species—has demonstrated broad pharmacological properties across multiple cancer models.

Gastric cancer (GC) remains one of the most common and lethal malignancies of the digestive system worldwide (Bray et al. 2024). Although advances in surgery, chemotherapy, targeted therapy, and immunotherapy have improved the management of GC, the prognosis of patients with advanced disease remains unsatisfactory (Burz et al. 2024). The progression of GC is driven by multiple interconnected biological processes, including uncontrolled proliferation, invasion, metastasis, angiogenesis, immune escape, and remodeling of the tumor microenvironment. These features highlight the complex and multifactorial nature of GC and underscore the need to identify effective therapeutic targets and multi‐pathway treatment strategies (Yasuda and Wang 2024).

Icariin, a major flavonoid glycoside isolated from Epimedium species, has been reported to possess anti‐inflammatory, antioxidant, immunomodulatory, and antitumor properties (Bi et al. 2022; Liu et al. 2023). Previous studies have shown that icariin can regulate tumor cell proliferation, apoptosis, migration, invasion, and angiogenesis in several cancers. In gastric cancer, icariin has also been suggested to affect malignant biological behaviors (Wang et al. 2010; Zhang et al. 2022). However, as a natural compound with multi‐target characteristics, its key targets and underlying molecular mechanisms in GC have not been systematically clarified (Liu et al. 2023).

Network pharmacology provides a systems‐level approach to explore the “multi‐target and multi‐pathway” effects of natural compounds (Zhang et al. 2013; Zhou et al. 2020). Recent advances have further emphasized the integration of multi‐omics data and experimental validation to improve the reliability and reproducibility of network pharmacology studies (Panossian 2025; Zhai et al. 2025). By integrating drug target prediction, disease‐related gene screening, functional enrichment analysis, and interaction network construction, this strategy is particularly suitable for elucidating the pharmacological mechanisms of bioactive compounds derived from traditional Chinese medicine. Therefore, network pharmacology offers an effective framework for understanding the potential therapeutic effects of icariin in gastric cancer (Zhang et al. 2013; Zhou et al. 2020).

In the present study, we combined network pharmacology, bulk transcriptomic analysis, and single‐cell RNA sequencing analysis to systematically investigate the potential mechanisms of icariin in GC. Potential targets of icariin and GC‐related genes were identified from public databases, and overlapping targets were further screened. Bulk transcriptomic data were then used for prognostic evaluation and functional enrichment analysis, while single‐cell RNA‐seq data were applied to characterize the expression patterns and potential biological significance of relevant genes in different cell populations within the tumor microenvironment. This study may further provide a theoretical basis for the development of icariin‐based functional food strategies targeting NOX4/ROS signaling in the prevention of gastric cancer as an NCCD.

## 2 Materials and Methods

### 2.1 Data Collection

Bulk transcriptomic data and corresponding clinical information were obtained from the TCGA and GEO databases. The TCGA‐STAD cohort was used as the primary cohort. GEO datasets GSE15497, GSE84437, and GSE84433 were included as external gastric cancer transcriptomic validation cohorts, while GSE26253 was included as an immunotherapy relapse/non‐relapse cohort. Single‐cell transcriptomic data were obtained from the GEO dataset GSE183904, which contains 40 gastric tissue samples, including 29 tumor samples and 11 normal samples.

Potential targets of icariin were retrieved from the TCMSP database, and target names were converted into official gene symbols using the UniProt database. Gastric cancer‐related genes were defined as the union of differentially expressed genes and WGCNA‐derived genes from modules significantly associated with gastric cancer in the TCGA‐STAD cohort. The intersection between icariin targets and gastric cancer‐related genes was identified using the Venn Diagram package in R, and a Venn diagram was generated.

### 2.2 Differential Expression Analysis

Differential expression analysis between tumor and normal tissues in the TCGA‐STAD cohort was performed using the limma package in R. Genes with |log2 fold change| > 1 and adjusted *p* < 0.05 were considered differentially expressed genes. The results were visualized using volcano plots.

### 2.3 Weighted Gene Co‐Expression Network Analysis

Weighted gene co‐expression network analysis was performed using the WGCNA package in R (Langfelder and Horvath 2008). The optimal soft‐thresholding power was determined based on the scale‐free topology fit index and mean connectivity. Gene modules were identified using the dynamic tree cut method, and hierarchical clustering was used to construct the module dendrogram. Pearson correlation analysis was then performed between module eigengenes and clinical traits to identify modules significantly associated with gastric cancer.

### 2.4 Survival Analysis of NOX4

Patients were divided into high‐ and low‐NOX4 expression groups according to the median NOX4 expression level. Overall survival was analyzed using the Kaplan–Meier method, and differences between groups were assessed by the log‐rank test. External validation of the prognostic value of NOX4 was further performed in the GSE15497, GSE84437, and GSE26253 cohorts.

### 2.5 Nomogram Construction and Validation

A prognostic nomogram was constructed based on the TCGA‐STAD cohort using the rms package in R by integrating relevant clinicopathological variables. The nomogram was used to predict 1‐, 3‐, and 5‐year overall survival probabilities. Calibration curves were generated to evaluate the agreement between predicted and observed survival outcomes.

### 2.6 Processing and Annotation of Single‐Cell RNA‐Seq Data

Single‐cell RNA sequencing data were processed using the Seurat package in R (Gulati et al. 2020). Seurat objects were first generated using the CreateSeuratObject function, and mitochondrial gene content was calculated using PercentageFeatureSet. Potential doublets were identified and removed using the DoubletFinder package. Quality control was then performed to retain high‐quality cells with more than 200 detected genes and less than 15% mitochondrial gene content. The data were normalized using NormalizeData, and highly variable genes were identified using FindVariableFeatures. After scaling with ScaleData, principal component analysis was performed using RunPCA. Batch effects across samples were corrected using the Harmony algorithm. Cells were then clustered using FindNeighbors and FindClusters, and visualized using RunUMAP. Marker genes for each cluster were identified using FindAllMarkers, and cell types were annotated according to canonical marker genes.

### 2.7 Copy Number Variation Inference and Malignant Cell Identification

Large‐scale copy number variation in epithelial cells was inferred from single‐cell transcriptomic data using the inferCNV (https://github.com/broadinstitute/inferCNV) package in R. Epithelial cells derived from normal samples were used as the reference, and copy number changes in epithelial cells from tumor tissues were inferred based on gene expression patterns. According to the inferCNV results, the mean square deviation was used as the measurement index, and the 90th percentile of normal epithelial cells was used as the cutoff to classify epithelial cells into high‐CNV and low‐CNV groups.

### 2.8 CytoTRACE Analysis

Cell differentiation status was evaluated using the CytoTRACE algorithm (Gulati et al. 2020). CytoTRACE predicts the relative differentiation state of each cell based on gene counts and expression levels. A higher CytoTRACE score indicates a less differentiated state, whereas a lower score indicates a more differentiated state.

### 2.9 Cell–Cell Communication Analysis

Cell–cell communication analysis was performed using the CellChat (Jin et al. 2025) package in R to infer ligand‐receptor interaction networks among different cell types. Outgoing and incoming interaction strengths were first calculated to assess the overall communication roles of each cell type in the tumor microenvironment. Interactions between NOX4pos_Endo and NOX4neg_Endo cells and other microenvironmental cell types, including fibroblasts, macrophages, monocytes, myofibroblasts, malignant cells, and normal epithelial cells, were then compared. Significant ligand‐receptor interactions were visualized using bubble plots.

### 2.10 Functional Enrichment Analysis

Functional enrichment analyses, including GSEA, GO enrichment, KEGG enrichment, and GSVA, were performed using the clusterProfiler (Wu et al. 2021) and GSVA (Hänzelmann et al. 2013) packages in R. GSEA was conducted based on differential gene expression profiles using the KEGG pathway database as the reference gene set, and pathways with |NES| > 1 and FDR < 0.25 were considered significant. Differentially expressed genes were further subjected to GO and KEGG enrichment analyses. In addition, GSVA was used to calculate pathway activity scores at the sample level.

### 2.11 Transcription Factor Regulatory Network Analysis

Transcription factor regulatory network analysis was performed using the SCENIC package in R (Aibar et al. 2017). Regulon activity was assessed to identify active transcription factors in different endothelial cell subgroups.

### 2.12 Metabolic Pathway Analysis

Metabolic pathway activity was evaluated using the scMetabolism R package (Wu et al. 2022). The relative activities of metabolic pathways were compared between endothelial cell subgroups and visualized using heatmaps.

### 2.13 Prognostic Validation of the NOX4pos_Endo Signature

Differentially expressed feature genes of the NOX4pos_Endo subgroup were extracted, and single‐sample gene set enrichment analysis was performed to calculate the NOX4pos_Endo score for each sample in the TCGA‐STAD, GSE84437, and GSE84433 cohorts. Patients were divided into high‐ and low‐score groups according to the median score, and survival differences between groups were evaluated using Kaplan–Meier analysis and the log‐rank test.

### 2.14 Consensus Clustering Based on the NOX4pos_Endo Signature

Consensus clustering was performed using the Consensus Cluster Plus package in R (Wilkerson and Hayes 2010) to identify molecular subtypes of gastric cancer. Samples were clustered using the K‐means algorithm with squared Euclidean distance, and the number of clusters (k) was tested from 2 to 9. The optimal number of clusters was determined according to the cumulative distribution function curves.

### 2.15 Construction and Evaluation of the NOX4‐Positive Endothelial Cell‐Related Prognostic Model

Shared candidate genes from the TCGA‐STAD, GSE84437, and GSE84433 cohorts were first subjected to univariate Cox regression analysis to identify prognosis‐related genes. Genes with prognostic significance in all three cohorts were retained for subsequent model construction. LASSO‐Cox regression was then performed using the glmnet package in R, and the optimal penalty parameter was determined by cross‐validation. A prognostic risk model was established based on the selected genes.

The risk score for each patient was calculated as follows: Risk score = Σ (Coefᵢ × Expᵢ) Patients were divided into high‐ and low‐risk groups according to the median risk score in each cohort. Survival differences between groups were assessed using Kaplan–Meier analysis and the log‐rank test. Time‐dependent ROC curves were generated using the timeROC package to evaluate the predictive performance of the model for 1‐, 3‐, and 5‐year overall survival. Univariate and multivariate Cox regression analyses were further performed to determine whether the risk score was an independent prognostic factor.

### 2.16 Immune Microenvironment Evaluation

The ESTIMATE algorithm (Yoshihara et al. 2013) was used to calculate Stromal Score, Immune Score, and Tumor Purity in the TCGA‐STAD cohort, and differences between high‐ and low‐risk groups were compared. In addition, the MCPcounter algorithm (Becht et al. 2016) was used to quantify the infiltration levels of immune and stromal cell populations, including cytotoxic lymphocytes, endothelial cells, fibroblasts, NK cells, and T cells.

### 2.17 Molecular Docking and Molecular Dynamics Simulation

The three‐dimensional structure of icariin was obtained from the PubChem database (PubChem CID: 5318997) and optimized using Chem3D 20.0. The predicted three‐dimensional structure of human NADPH oxidase 4 (NOX4) was retrieved from the RCSB Protein Data Bank (computed structure model ID: AF_AFQ9NPH5F1). After routine receptor and ligand preparation, molecular docking was performed using BIOVIA Discovery Studio 2019 and AutoDock Vina 1.2.3. Representative docking poses and predicted amino acid interactions were visualized using PyMOL 2.5.3.

The selected icariin–NOX4 complex was subsequently subjected to a 100‐ns molecular dynamics simulation using GROMACS 2019.6. Following energy minimization and NVT/NPT equilibration, the stability of the complex was evaluated using RMSD analysis and representative conformational superposition.

### 2.18 Cell Culture

The human gastric adenocarcinoma cell line AGS was cultured in Ham's F‐12 K (Kaighn's modification) medium supplemented with 10% fetal bovine serum (FBS) and 1% penicillin–streptomycin. Cells were maintained at 37°C in a humidified incubator with 5% CO2. Cells in logarithmic growth phase were used for all experiments. Mycoplasma contamination was routinely tested and confirmed negative throughout the study.

### 2.19 RNA Extraction and RT‐qPCR

Total RNA was extracted from cells using a commercial RNA extraction kit (Servicebio, cat. no. G3640) according to the manufacturer's instructions. RNA purity and concentration were assessed using a Nanodrop 2000 spectrophotometer (Thermo Fisher Scientific). Complementary DNA (cDNA) was synthesized from 1 μg of total RNA using the PrimeScript RT Reagent Kit (TAKARA, cat. no. RR037A) following the manufacturer's protocol. The resulting cDNA was diluted to a final concentration of 200 ng/μL prior to use.

Quantitative real‐time PCR (qPCR) was carried out using the SYBR Green‐based fluorescence detection system (Vazyme, cat. no. Q711) on a real‐time PCR instrument. Each reaction was performed in a total volume of 20 μL. The thermal cycling conditions were as follows: initial denaturation at 95°C for 30 s, followed by 40 cycles of 95°C for 10 s and 60°C for 30 s. GAPDH was used as an internal reference gene, and relative mRNA expression levels were calculated using the 2 − ^ΔΔCT^ method. The primer sequences used in this study are listed in Table 1.

**TABLE 1:** Primer sequences used for RT‐qPCR.

Gene	Forward primer (5′ → 3′)	Reverse primer (5′ → 3′)
h‐NOX4	GCCAGAGTATCACTACCTCCAC	CTCGGAGGTAAGCCAAGAGTGT
h‐GAPDH	CAGGAGGCATTGCTGATGAT	GAAGGCTGGGGCTCATTT

### 2.20 CCK‐8 Assay for IC50 Determination

Cell viability was assessed using the CCK‐8 assay. Cells were seeded in 96‐well plates at a density of 5 × 103 cells per well and allowed to adhere overnight. The culture medium was then replaced with medium containing icariin at concentrations of 0, 10, 20, 40, 80, 160, and 320 μM, and cells were incubated for 24 h. Subsequently, 10 μL of CCK‐8 reagent was added to each well, and the plates were incubated at 37°C for 2 h. Absorbance at 450 nm was measured using a microplate reader. Cell viability was plotted against drug concentration, and IC50 values were calculated by nonlinear regression fitting using GraphPad Prism 10 software. 6 replicate wells were included per group, and the experiment was repeated three times independently.

### 2.21 Intracellular ROS Detection

After the indicated treatments, cells were washed twice with serum‐free culture medium. The DCFH‐DA fluorescent probe (Elabscience, cat. no. E‐BC‐K138‐F) was diluted 1:1000 in serum‐free medium, and 1 mL of the working solution was added to each well of a six‐well plate. Plates were wrapped in aluminum foil and incubated at 37°C for 1 h. Cells were then washed three times with serum‐free DMEM, replenished with complete culture medium, and immediately examined by fluorescence microscopy. Intracellular ROS levels were evaluated by semi‐quantitative fluorescence image analysis.

### 2.22 Western Blot Analysis

Total protein was extracted from cells using RIPA lysis buffer supplemented with a protease inhibitor cocktail, and protein concentration was determined by the BCA protein assay. Equal amounts of protein were resolved by SDS‐PAGE and transferred onto PVDF membranes. Membranes were blocked with 5% non‐fat dried milk in TBST for 2 h at room temperature, followed by incubation with the corresponding primary antibodies overnight at 4°C. After washing 3 times with TBST, membranes were incubated with HRP‐conjugated secondary antibodies for 1 h at room temperature. Protein bands were visualized by ECL chemiluminescence. GAPDH was used as the loading control, and band intensities were quantified using ImageJ software. The following primary antibodies were used: anti‐NOX4 (cat. no. 14347–1‐AP, Proteintech, rabbit polyclonal, 1:1000).

### 2.23 Statistical Analysis

All statistical analyses were performed using R software (version 4.3). For comparisons between two independent groups, the Wilcoxon rank‐sum test was applied to non‐normally distributed continuous variables, whereas Student's *t*‐test was used when data satisfied the assumptions of normality and homogeneity of variance. Comparisons across three or more groups were performed using the Kruskal‐Wallis test, with post hoc pairwise testing applied when overall significance was detected. Survival analysis was performed using the Kaplan–Meier method, and between‐group differences in survival distributions were evaluated using the log‐rank test. Univariate and multivariate Cox proportional hazards regression models were constructed to assess the independent prognostic significance of variables of interest. Prognostic model performance was evaluated by time‐dependent receiver operating characteristic (ROC) analysis using the timeROC package, and the area under the curve (AUC) was reported for 1‐, 3‐, and 5‐year overall survival. For in vitro experimental data, results are presented as mean ± standard deviation (SD) from at least three independent experiments. Multiple‐group comparisons in cell‐based experiments were performed using one‐way analysis of variance (ANOVA) followed by Dunnett's multiple comparisons test against the control group. Unless stated otherwise, all statistical tests were two‐sided, and a *p*‐value < 0.05 was considered statistically significant.

## 3 Results

### 3.1 Screening of Key Genes in Gastric Cancer and Identification of Intersecting Targets of Icariin

Differential expression analysis revealed marked transcriptional differences between gastric cancer and normal tissues, identifying a set of significantly dysregulated genes (Figure 1A). Subsequently, a weighted gene co‐expression network was constructed using WGCNA, and multiple co‐expression modules were identified after selecting the optimal soft‐thresholding power (Figure 1B,C). Module‐trait relationship analysis showed that the turquoise module was most strongly associated with the gastric cancer phenotype and was therefore selected as the key module (Figure 1D). KEGG enrichment analysis indicated that genes in the turquoise module were mainly enriched in pathways related to cell cycle, DNA replication, p53 signaling, and viral carcinogenesis (Figure 1E). Key genes associated with gastric cancer were then intersected with the putative targets of icariin, yielding 24 overlapping targets (Figure 1F). Univariate Cox regression analysis further showed that NOX4 was significantly associated with patient prognosis, suggesting that it may represent a potential target associated with the effects of icariin in gastric cancer (Figure 1G).

**FIGURE 1:**
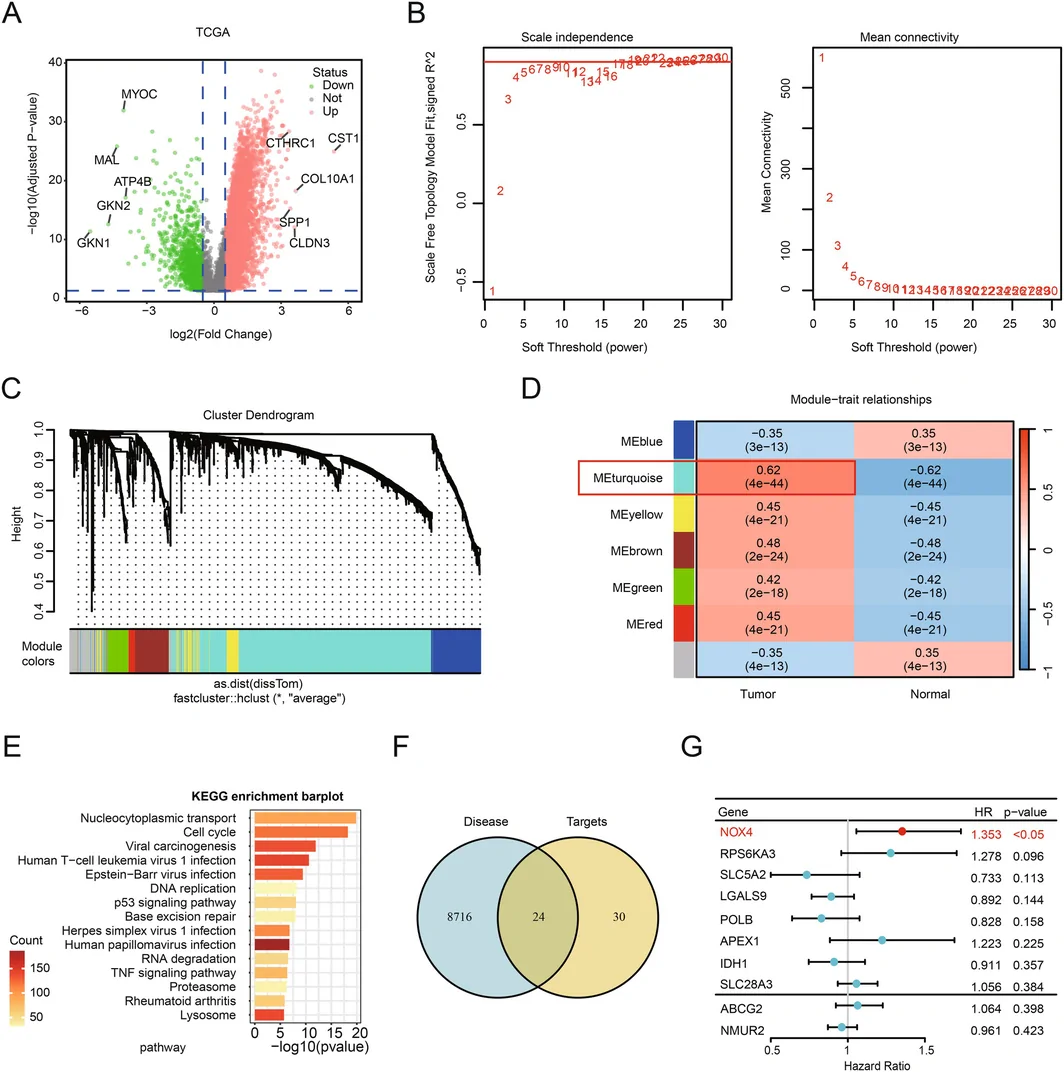
Screening of key genes in gastric cancer and identification of intersecting targets of Icariin. (A) Volcano plot of differentially expressed genes in gastric cancer. (B) Soft‐threshold analysis in WGCNA. (C) Gene clustering dendrogram and module identification of co‐expression modules. (D) Heatmap of module‐trait relationships. (E) KEGG enrichment analysis of genes in the key turquoise module. (F) Intersection analysis between gastric cancer‐related genes and putative targets of icariin. (G) Univariate Cox regression analysis of the intersecting targets.

### 3.2 Analysis of Aberrant Expression, Prognostic Value, and Functional Characteristics of NOX4 in Gastric Cancer

To further clarify the biological significance of NOX4 in gastric cancer, its expression pattern was first investigated. Pan‐cancer analysis showed that NOX4 was aberrantly expressed in multiple tumor types and was markedly upregulated in gastric cancer (Figure 2A). Given its dysregulated expression in gastric cancer, the prognostic relevance of NOX4 was further evaluated in three independent cohorts, including GSE15497, GSE84437, and GSE26253. The results consistently showed that high NOX4 expression was significantly associated with poor prognosis, suggesting that NOX4 may have relatively consistent prognostic relevance across cohorts (Figure 2B). To explore the potential molecular features associated with NOX4, GSEA was performed based on the TCGA cohort. The high‐NOX4 expression group was mainly enriched in tumor progression‐related pathways, including ECM‐receptor interaction, pathways in cancer, focal adhesion, and the TGF‐beta signaling pathway (Figure 2C), whereas the low‐NOX4 expression group was primarily enriched in metabolism‐related pathways, such as fatty acid degradation, pyruvate metabolism, drug metabolism‐cytochrome P450, and retinol metabolism (Figure 2F). Further clinicopathological analysis showed that NOX4 expression was significantly associated with pathological grade and clinical stage in gastric cancer, with an overall increasing trend along disease progression (Figure 2D,E). Based on these observations, a nomogram integrating NOX4 expression and clinical characteristics was constructed to further evaluate its potential prognostic utility, and the calibration curves showed good agreement between the predicted and observed outcomes (Figure 2G,H).

**FIGURE 2:**
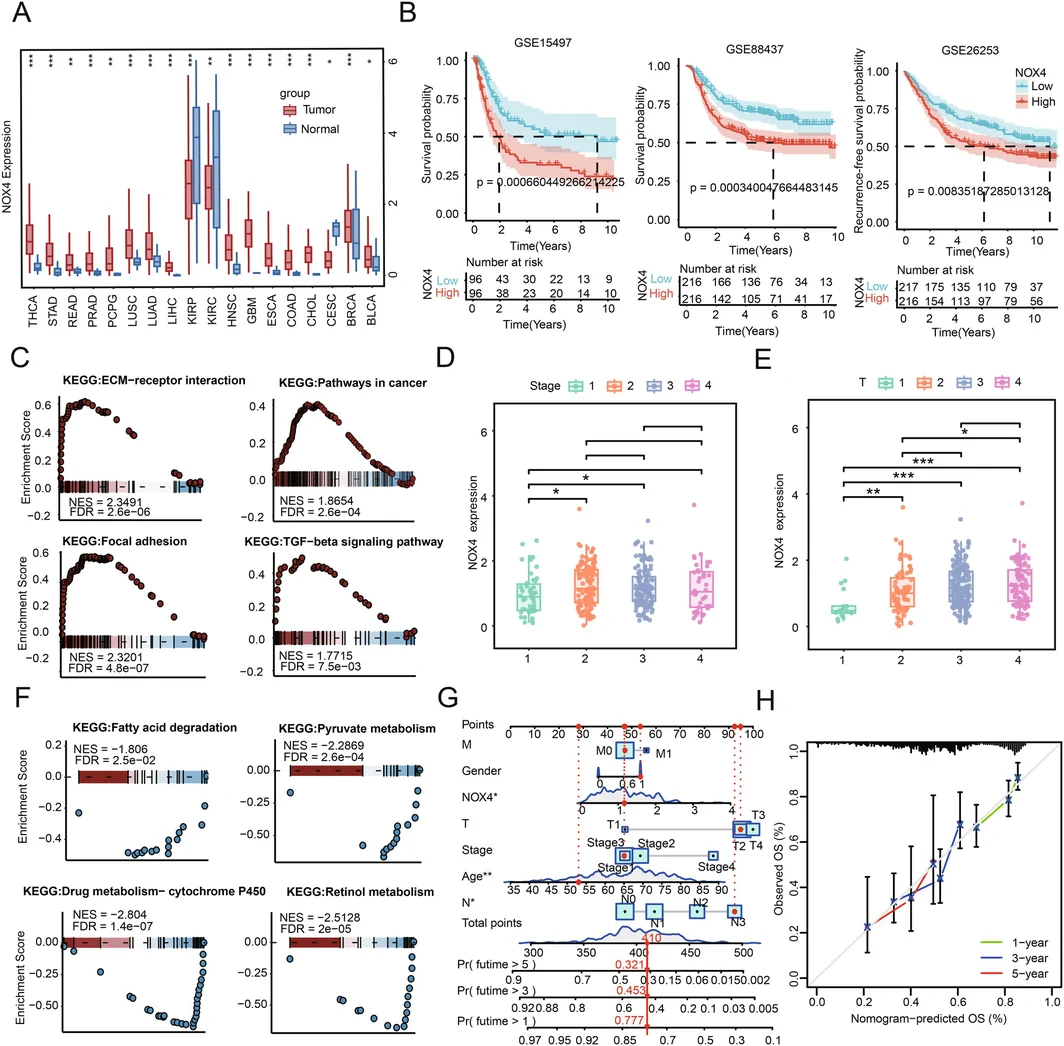
Expression pattern, multi‐cohort prognostic value, and functional analysis of NOX4. (A) Pan‐cancer expression profile of NOX4. (B) Survival analyses of NOX4 in the GSE15497, GSE84437, and GSE26253 cohorts. (C) GSEA of the high‐NOX4 expression group in the TCGA cohort. (D) Association between NOX4 expression and pathological grade in gastric cancer. (E) Association between NOX4 expression and clinical stage in gastric cancer. Statistical significance was assessed using the Kruskal–Wallis test followed by post hoc pairwise comparisons.*p < 0.05, **p < 0.01, ***p < 0.001. (F) GSEA of the low‐NOX4 expression group in the TCGA cohort. (G) Nomogram integrating NOX4 expression and clinical characteristics. (H) Calibration curves of the nomogram for overall survival prediction.

### 3.3 Single‐Cell Transcriptomic Analysis Reveals Cellular Heterogeneity and Malignant Epithelial Features in Gastric Cancer

To characterize cellular heterogeneity and the tumor microenvironment of gastric cancer at single‐cell resolution, an integrated analysis was performed on single‐cell transcriptomic data from 40 samples, including 29 tumor samples and 11 normal samples, comprising a total of 142,053 cells. After quality control, Harmony‐based batch correction, dimensionality reduction, and clustering, cells from different samples were well integrated and classified into 38 clusters (Figure S1). Based on the expression patterns of canonical marker genes, these cells were further annotated into 11 major cell populations, including T/NK cells, malignant/epithelial cells, plasma cells, macrophages, endothelial cells, myofibroblasts, B cells, monocytes, fibroblasts, mast cells, and dendritic cells (Figure 3A–C).

**FIGURE 3:**
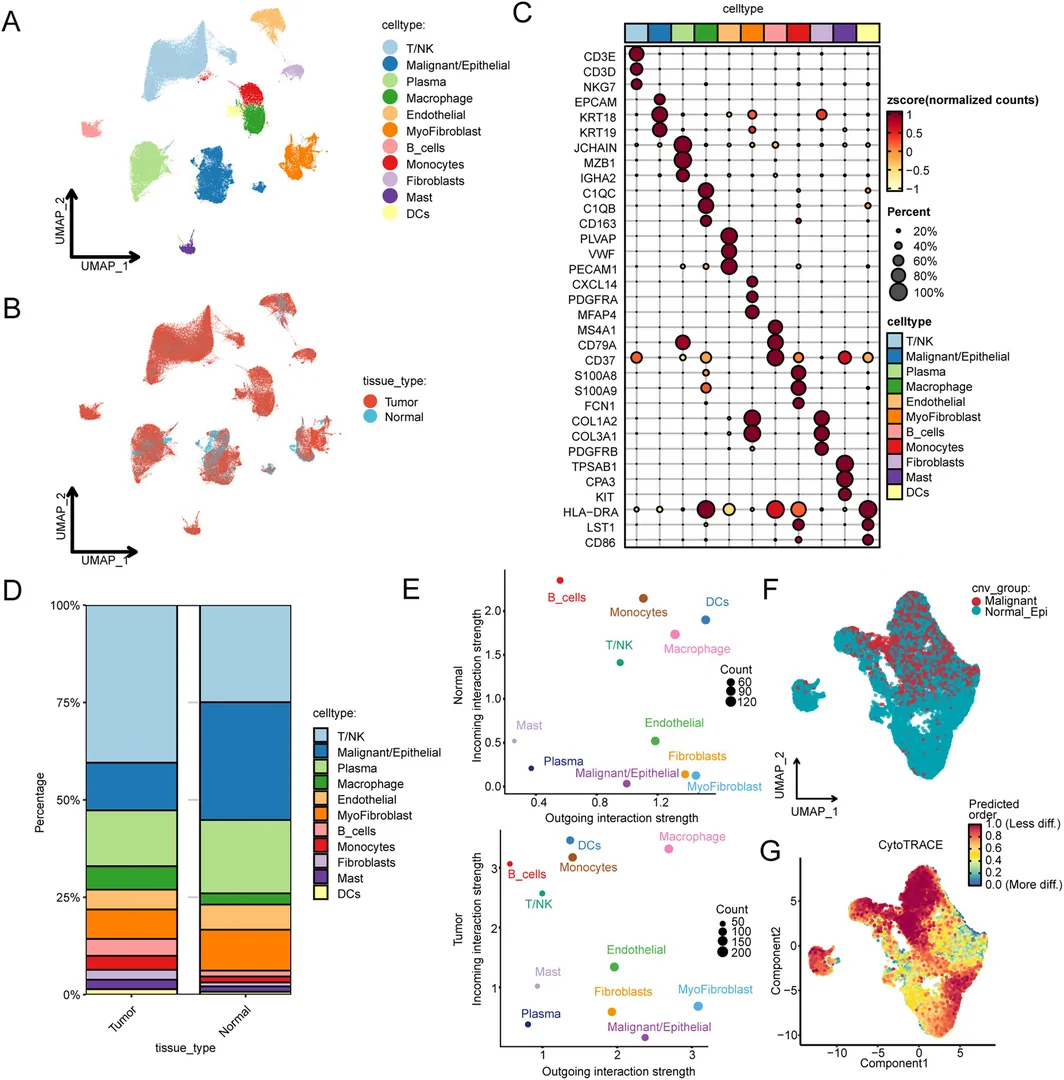
Single‐cell transcriptomic landscape of gastric cancer reveals differences in cellular composition, cell–cell communication, and malignant‐associated cellular states. (A) UMAP visualization of annotated cell populations in the gastric cancer single‐cell transcriptomic dataset. (B) Distribution of cell populations in tumor and normal tissues. (C) Heatmap showing the expression of canonical marker genes across annotated cell populations. (D) Differences in cellular composition between tumor and normal tissues. (E) Analysis of intercellular communication strength. (F) Distribution of cells based on CNV grouping. (G) CytoTRACE analysis showing the differentiation potential of different cell populations.

Comparison of cellular distributions between tumor and normal tissues showed marked differences in the representation of individual cell populations (Figure 3B). In particular, malignant/epithelial cells were markedly enriched in tumor tissues, accompanied by substantial remodeling of several immune and stromal populations (Figure 3D), suggesting pronounced compositional heterogeneity within the gastric cancer microenvironment. To further characterize the epithelial compartment, epithelial cells were extracted and reclustered, followed by inferCNV analysis. The results showed that epithelial subpopulations exhibited distinct CNV patterns and could be further classified into high‐CNV and low‐CNV groups, suggesting the presence of epithelial subsets with malignant‐associated CNV features and considerable intratumoral heterogeneity (Figure S2).

Following the identification of major cell populations and malignant epithelial subsets, intercellular communication within the gastric cancer microenvironment was further investigated. Cell–cell communication analysis showed extensive and complex interactions among different cell types, with myeloid populations such as macrophages, monocytes, and dendritic cells displaying relatively strong communication activity (Figure 3E). In addition, the distribution of cells according to CNV grouping showed distinct patterns in the low‐dimensional space (Figure 3F), further supporting malignant‐associated heterogeneity within the gastric cancer cell populations. CytoTRACE analysis demonstrated different differentiation potentials across cell populations, with some cells exhibiting a less differentiated state (Figure 3G), suggesting that the gastric cancer microenvironment exhibits heterogeneity not only in cellular composition but also in cellular states.

### 3.4 Functional Features, Cell–Cell Communication, and Prognostic Relevance of NOX4‐Positive Endothelial Cells

To further explore the cellular source and potential functional relevance of NOX4 in the gastric cancer microenvironment, NOX4 expression was examined at the single‐cell level. NOX4 was mainly expressed in endothelial cells, whereas its expression was relatively limited in other cell populations, suggesting that endothelial cells may represent a major cellular context for NOX4‐related activity (Figure 4A). Based on this observation, endothelial cells were further stratified into NOX4‐positive endothelial cells (NOX4pos_Endo) and NOX4‐negative endothelial cells (NOX4neg_Endo), which showed partially distinct distributions in the low‐dimensional space, indicating potential transcriptional differences between the two subsets (Figure 4B).

**FIGURE 4:**
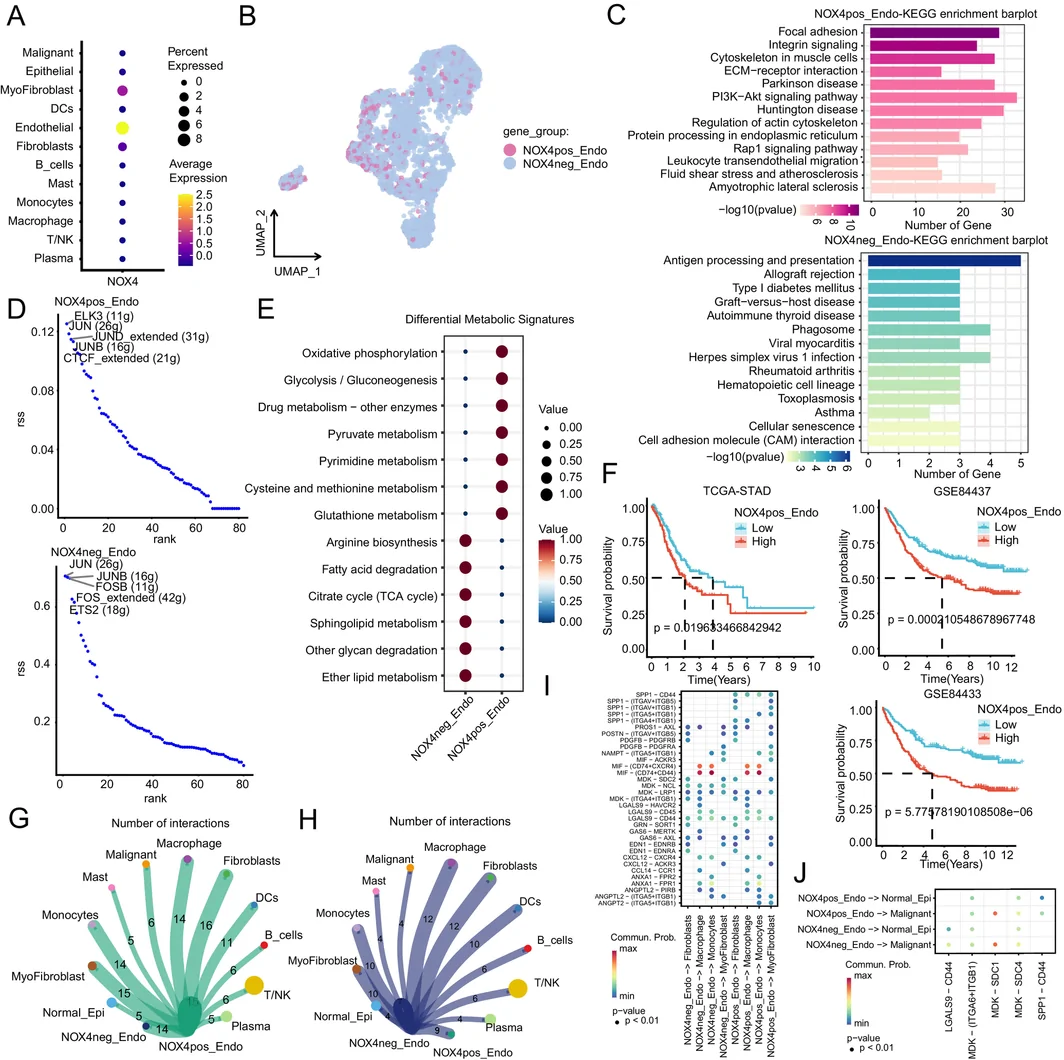
Functional characteristics, cell–cell communication, and prognostic relevance of NOX4‐positive endothelial cells. (A) Expression distribution of NOX4 across different cell types. (B) UMAP distribution of NOX4‐positive endothelial cells (NOX4pos_Endo) and NOX4‐negative endothelial cells (NOX4neg_Endo). (C) KEGG enrichment analysis comparing NOX4pos_Endo and NOX4neg_Endo. (D) Ranking of key transcription factor activities in NOX4pos_Endo and NOX4neg_Endo based on SCENIC analysis. (E) Differential metabolic pathway analysis between NOX4pos_Endo and NOX4neg_Endo. (F) Survival analysis of the NOX4pos_Endo‐associated gene signature in the TCGA‐STAD, GSE84437, and GSE84433 cohorts. (G) Number of cell–cell interactions between NOX4pos_Endo and other cell populations. (H) Number of cell–cell interactions between NOX4neg_Endo and other cell populations. (I) Significant ligand‐receptor interactions of NOX4‐positive and NOX4‐negative endothelial cells with different cell populations. (J) Representative ligand‐receptor interactions between NOX4‐positive or NOX4‐negative endothelial cells and normal epithelial or malignant cells.

Functional enrichment analysis suggested that NOX4pos_Endo was mainly associated with pathways related to cell adhesion, matrix remodeling, and angiogenesis, including focal adhesion, integrin signaling, ECM‐receptor interaction, the PI3K‐Akt signaling pathway, and regulation of the actin cytoskeleton. By contrast, NOX4neg_Endo appeared to be more associated with immune‐related pathways, such as antigen processing and presentation, allograft rejection, and phagosome (Figure 4C). Transcription factor analysis further indicated relatively higher activities of ELK3, JUN, and JUNB in NOX4pos_Endo, whereas JUN, FOSB, and ETS2 were more enriched in NOX4neg_Endo (Figure 4D). In addition, metabolic pathway analysis suggested that NOX4pos_Endo was more associated with oxidative phosphorylation, glycolysis/gluconeogenesis, pyruvate metabolism, and glutathione metabolism, whereas NOX4neg_Endo showed relatively higher associations with arginine biosynthesis, fatty acid degradation, the citrate cycle, and sphingolipid metabolism (Figure 4E). Together, these findings suggest that NOX4‐positive endothelial cells may exhibit distinct functional and metabolic features.

The clinical relevance of the NOX4pos_Endo‐associated gene signature was further assessed in the TCGA‐STAD, GSE84437, and GSE84433 cohorts. Survival analysis showed that a high NOX4pos_Endo signature score was associated with poorer prognosis across all 3 cohorts (Figure 4F). Cell–cell communication analysis indicated that NOX4pos_Endo had broader interactions with multiple cell populations, particularly macrophages, fibroblasts, dendritic cells, and T/NK cells, whereas NOX4neg_Endo showed relatively fewer interactions overall (Figure 4G,H). Ligand‐receptor analysis further identified several potential interactions between NOX4‐positive endothelial cells and normal epithelial or malignant cells, involving signaling molecules such as SPP1, MDK, MIF, and ANGPTL2 (Figure 4I,J). These results suggest that NOX4‐positive endothelial cells may be associated with microenvironmental remodeling in gastric cancer.

### 3.5 Molecular Subtyping of Gastric Cancer Based on the NOX4‐Positive Endothelial Cell Signature

To further evaluate the stratification value of the NOX4‐positive endothelial cell‐associated signature in gastric cancer, consensus clustering was performed in the TCGA‐STAD cohort based on the NOX4pos_Endo‐related gene signature. The results suggested that *k* = 2 provided relatively stable clustering, and patients were therefore classified into 2 subtypes, Cluster1 and Cluster2 (Figure 5A,B). The heatmap showed that the 2 subtypes exhibited distinct expression patterns of NOX4pos_Endo‐associated genes and differed in several clinicopathological features (Figure 5C). In addition, the NOX4pos_Endo score was significantly higher in Cluster2 than in Cluster1 (Figure 5D), suggesting that Cluster2 was more strongly associated with the NOX4‐positive endothelial cell‐related signature.

**FIGURE 5:**
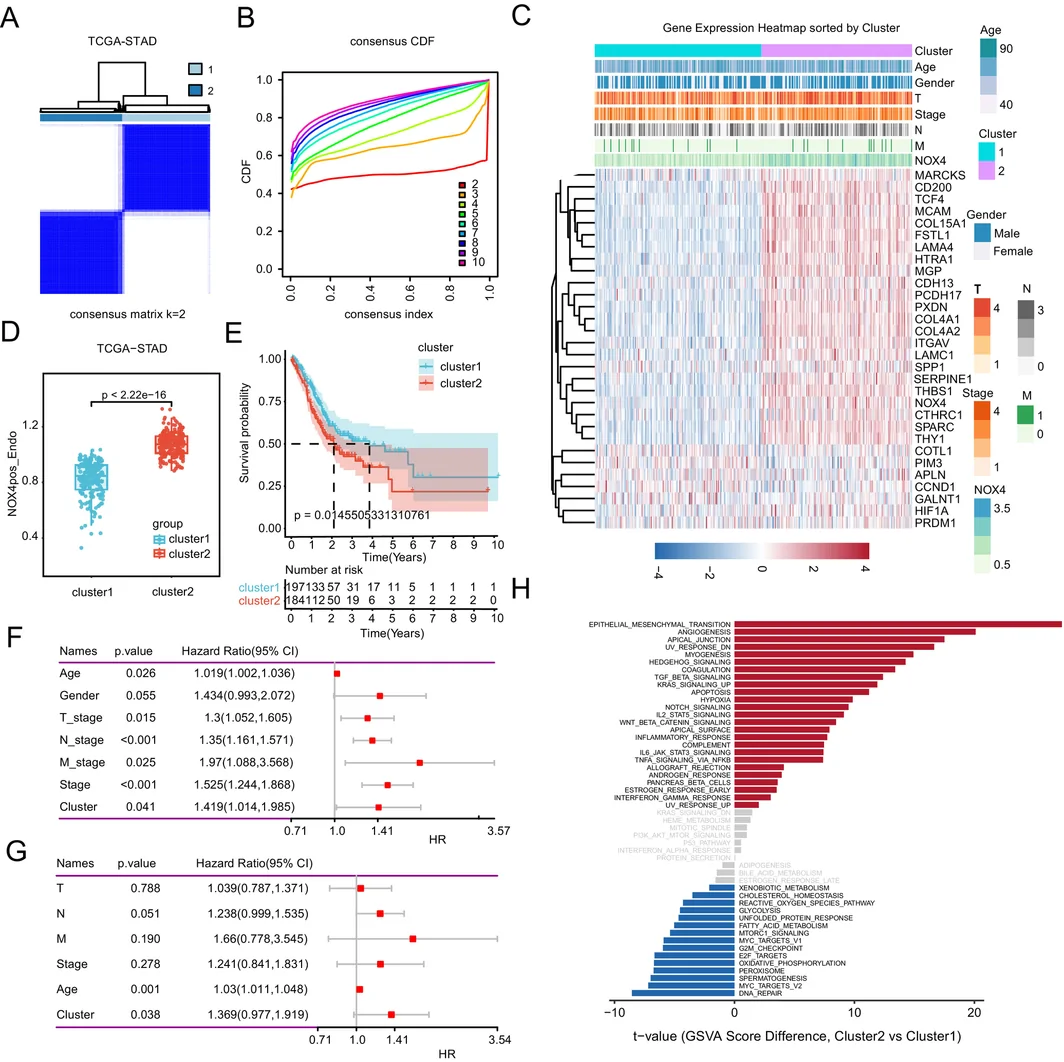
Molecular subtyping of gastric cancer based on the NOX4‐positive endothelial cell signature and its clinical relevance. (A) Consensus clustering matrix for the TCGA‐STAD cohort based on the NOX4‐positive endothelial cell‐associated gene signature (*k* = 2). (B) Cumulative distribution function (CDF) curves under different clustering numbers. (C) Heatmap showing gene expression patterns and clinical annotations of the two molecular subtypes. (D) Comparison of NOX4pos_Endo scores between the two subtypes. (E) Overall survival analysis of patients in the two subtypes. (F) Univariate Cox regression analysis. (G) Multivariate Cox regression analysis. (H) GSVA showing pathway differences between the two subtypes.

Survival analysis showed that patients in Cluster2 had a lower overall survival probability than those in Cluster1 (Figure 5E). Cox regression analyses further indicated that the subtype variable was associated with prognosis in both univariate and multivariate models (Figure 5F–G). GSVA further suggested that Cluster2 was relatively enriched in pathways related to epithelial‐mesenchymal transition, angiogenesis, apical junction, IL6‐JAK‐STAT3 signaling, TNFA signaling via NFkB, and inflammatory response, whereas Cluster1 was relatively more enriched in pathways including DNA repair, oxidative phosphorylation, peroxisome, MYC targets, fatty acid metabolism, and glycolysis (Figure 5H). In addition, immune infiltration analysis showed differences in the proportions of several immune cell populations between the two subtypes, including macrophages, regulatory T cells, and mast cells (Figure S3A,B). Multiple immune‐related molecules were also differentially expressed between Cluster1 and Cluster2 (Figure S3C). Taken together, these results suggest that the molecular subtypes defined by the NOX4‐positive endothelial cell signature may be associated with distinct biological characteristics, immune microenvironment features, and clinical outcomes in gastric cancer.

### 3.6 Cross‐Cohort Validation of NOX4pos_Endo‐Based Subtypes and Identification of Shared Gene Modules

To assess the robustness of the NOX4pos_Endo‐based subtyping across independent cohorts, consensus clustering was further performed in the GSE84437 and GSE84433 cohorts. In both datasets, *k* = 2 showed relatively stable clustering patterns, and Cluster2 consistently exhibited significantly higher NOX4pos_Endo scores and poorer overall survival than Cluster1 (Figure S4A–H), which was generally consistent with the findings in TCGA‐STAD. To further identify subtype‐associated gene modules, WGCNA was subsequently performed in the TCGA‐STAD, GSE84437, and GSE84433 cohorts. In all three datasets, the turquoise module showed the strongest association with Cluster2 (Figure S4I–K). Genes shared across these cluster‐associated modules were then extracted for downstream analysis, and KEGG enrichment suggested that they were mainly associated with focal adhesion, integrin signaling, ECM‐receptor interaction, and regulation of the actin cytoskeleton (Figure S4L). These shared genes were subsequently used as candidate features for prognostic model construction.

### 3.7 Construction of a NOX4‐Positive Endothelial Cell‐Related Prognostic Model and Analysis of Its Immune Microenvironment Features

To further evaluate the prognostic value of NOX4‐positive endothelial cell‐related genes, prognosis‐associated candidate genes shared by the GSE84437, GSE84433, and TCGA‐STAD cohorts were first identified by intersection analysis (Figure 6A). LASSO regression was then performed to reduce dimensionality and optimize variable selection, and the optimal penalty parameter was determined by cross‐validation (Figure 6B,C). Based on this procedure, a prognostic model was established using a subset of selected genes, including SPIRE1, PLCH1, KCNS3, SLC27A2, and GRP (Figure 6D).

**FIGURE 6:**
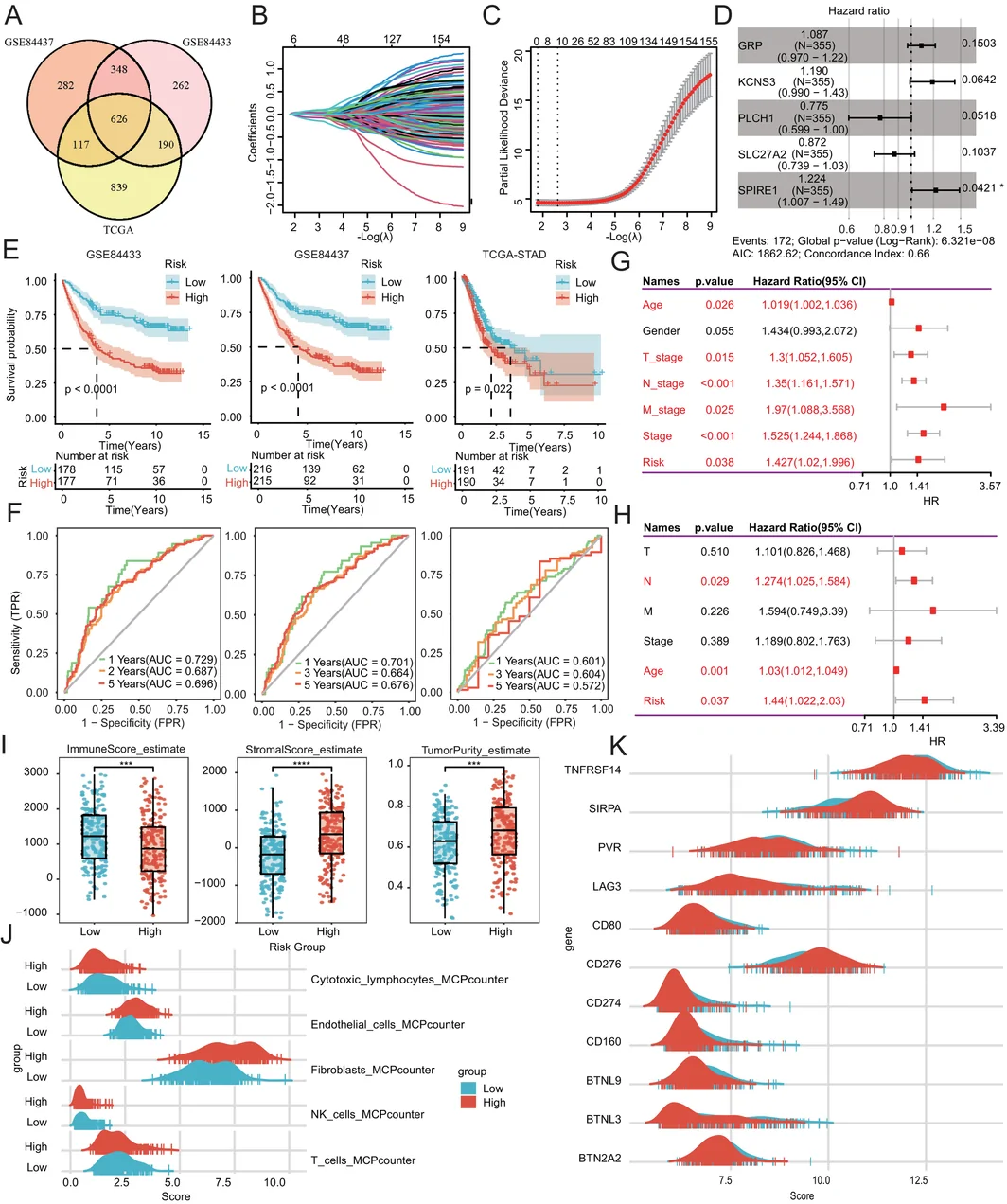
Construction, validation, and immune microenvironment characterization of the NOX4‐positive endothelial cell‐related risk model. (A) Intersection analysis of prognosis‐related candidate genes across the GSE84437, GSE84433, and TCGA‐STAD cohorts. (B) Coefficient trajectories of candidate genes under different penalty parameters in the LASSO regression analysis. (C) Cross‐validation for selection of the optimal penalty parameter in LASSO regression. (D) Forest plot of key genes included in the risk model. (E) Overall survival analysis of the risk score in the GSE84433, GSE84437, and TCGA‐STAD cohorts. (F) Time‐dependent ROC curves for predicting 1‐, 3‐, and 5‐year overall survival in the GSE84433, GSE84437, and TCGA‐STAD cohorts. (G) Univariate Cox regression analysis. (H) Multivariate Cox regression analysis. (I) Comparison of ImmuneScore, StromalScore, and TumorPurity between the low‐ and high‐risk groups. (J) Differential MCPcounter‐estimated immune/stromal cell infiltration between the low‐ and high‐risk groups. (K) Differential expression analysis of immune‐related molecules between the low‐ and high‐risk groups.

The prognostic performance of the model was subsequently evaluated in the GSE84433, GSE84437, and TCGA‐STAD cohorts. Survival analysis showed that patients in the high‐risk group had poorer overall survival than those in the low‐risk group across the three cohorts (Figure 6E). Time‐dependent ROC analysis further suggested that the risk model had moderate predictive performance for 1‐, 3‐, and 5‐year overall survival in these cohorts (Figure 6F). Cox regression analyses indicated that the risk score was associated with prognosis in both univariate and multivariate models (Figure 6G,H), suggesting that the model may provide additional prognostic information beyond conventional clinicopathological variables.

Importantly, the relationship between the 5‐gene risk model and the NOX4pos_Endo‐associated transcriptional program was examined independently of gene overlap. After excluding SPIRE1, PLCH1, KCNS3, SLC27A2, and GRP from the NOX4pos_Endo gene set, the recalculated signature score remained positively correlated with the risk score in the TCGA‐STAD, GSE84433, and GSE84437 cohorts (Figure S5A–C). These findings support the interpretation that the prognostic model captures broader transcriptional features associated with NOX4‐positive endothelial cells rather than being driven by direct gene overlap.

To further characterize biological differences between the two risk groups, tumor microenvironment‐related features were compared. The high‐risk group showed lower ImmuneScore, higher StromalScore, and higher TumorPurity than the low‐risk group (Figure 6I). MCPcounter analysis further suggested differences in several immune and stromal cell populations between the two groups, including cytotoxic lymphocytes, endothelial cells, fibroblasts, NK cells, and T cells (Figure 6J). In addition, multiple immune‐related molecules, such as TNFRSF14, SIRPA, PVR, LAG3, CD80, CD276, CD274, CD160, BTNL9, BTNL3, and BTN2A2, were differentially expressed between the 2 risk groups (Figure 6K). Taken together, these findings suggest that the NOX4‐positive endothelial cell‐related risk model may be associated not only with prognosis but also with distinct immune microenvironment features in gastric cancer.

### 3.8 Molecular Docking and Molecular Dynamics Simulation of Icariin With the Target Protein

To further evaluate the potential interaction between icariin and the target protein, molecular docking and molecular dynamics simulation were performed. The docking results suggested that icariin could be accommodated within the binding pocket of the target protein and form multiple interactions with surrounding residues (Figure 7A,B). The predicted binding mode indicated potential hydrogen‐bond interactions with residues such as Ser576 and Trp377, which may contribute to stabilization of the ligand–protein complex (Figure 7D).

**FIGURE 7:**
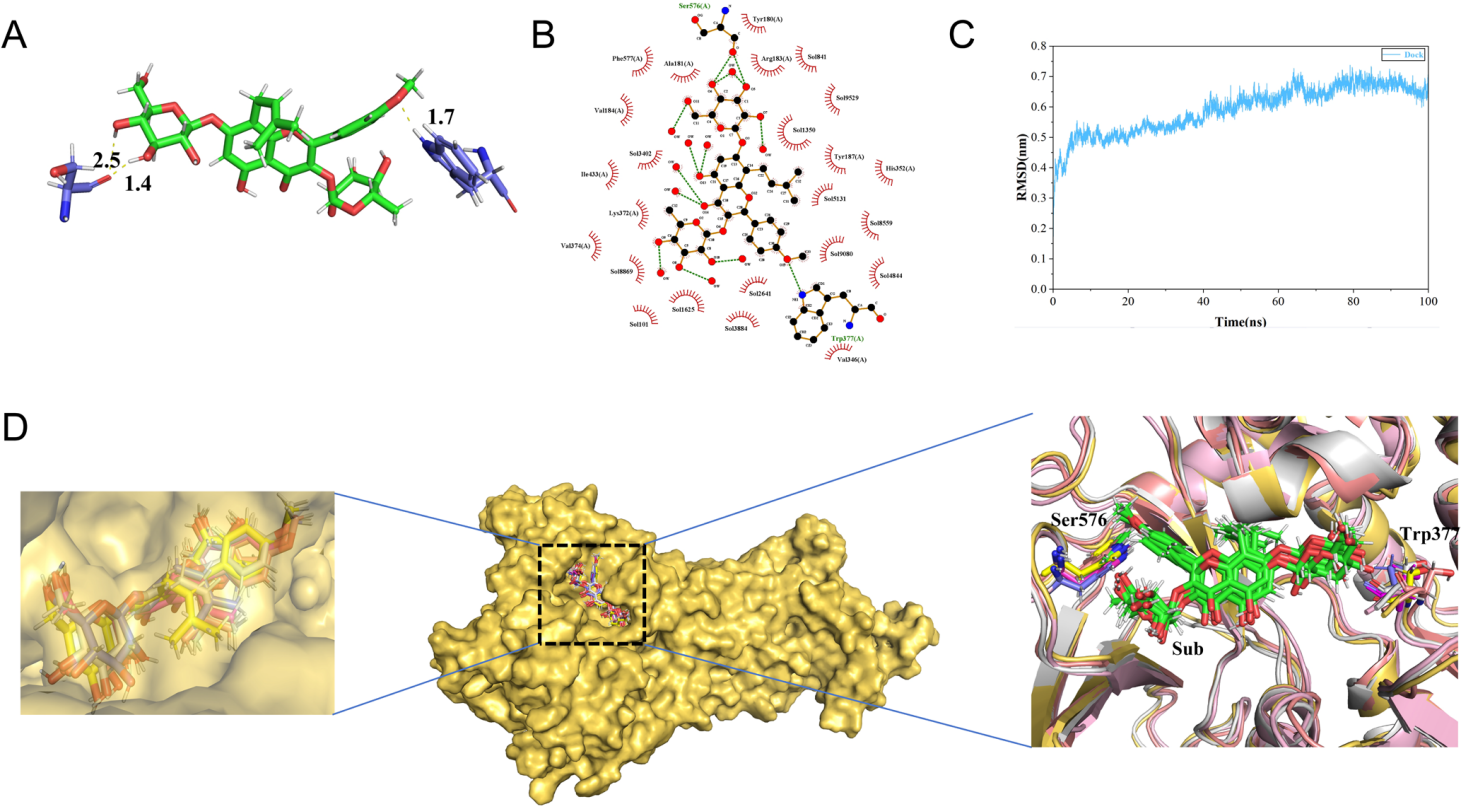
Molecular docking and molecular dynamics simulation of icariin with the target protein. (A) Three‐dimensional docking conformation of icariin within the binding pocket of the target protein. (B) Two‐dimensional schematic diagram of the interactions between icariin and surrounding amino acid residues. (C) RMSD trajectory of the icariin–protein complex during molecular dynamics simulation. (D) Superposition of representative conformations of the icariin–protein complex during the simulation, showing the binding mode of icariin within the pocket.

To further assess the stability of the docked complex, molecular dynamics simulation was conducted. The RMSD trajectory showed an initial fluctuation followed by a relatively stable plateau during the simulation period, suggesting that the icariin–protein complex remained generally stable over time (Figure 7C). In addition, superposition of conformations sampled during the simulation showed that icariin remained within the binding pocket with limited positional drift, further supporting the stability of the binding mode (Figure 7D). Taken together, these computational results support a plausible and relatively stable binding pose within the simulated system but do not establish direct physical binding between icariin and NOX4.

### 3.9 Experimental Validation of the Inhibitory Effect of Icariin on Cell Viability, NOX4 Expression, and Intracellular ROS Levels in AGS Cells

To experimentally evaluate the effect of icariin on gastric cancer cells, AGS cells were first treated with different concentrations of icariin, and cell viability was assessed. The results showed that icariin reduced cell viability in a concentration‐dependent manner, with an IC50 value of 124.7 μM (Figure 8A). Based on this result, subsequent experiments were performed using concentrations corresponding to IC25, IC50, and IC75.

**FIGURE 8:**
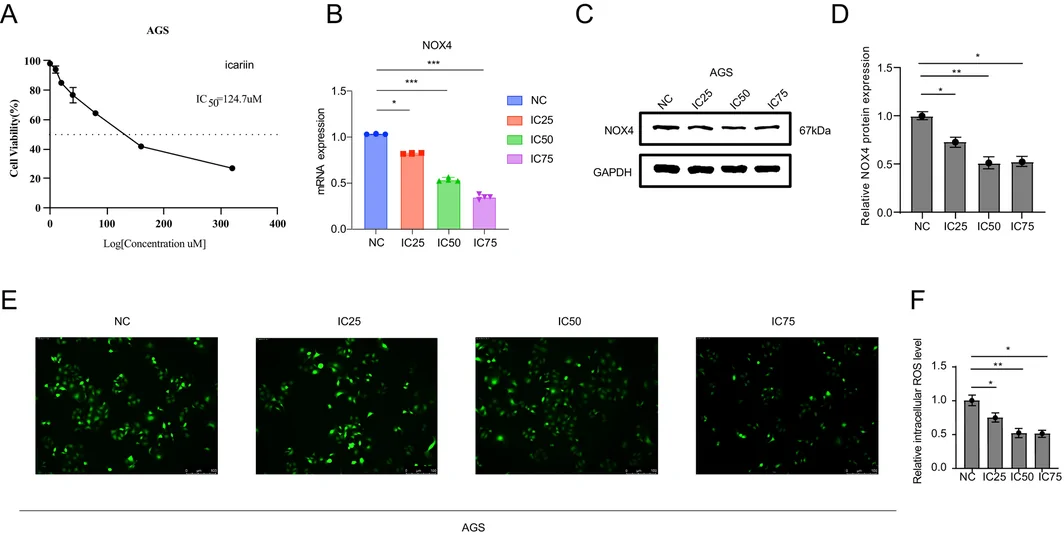
Experimental validation of the inhibitory effects of icariin on cell viability, NOX4 expression, and intracellular ROS levels in AGS cells. (A) Cell viability of AGS cells treated with different concentrations of icariin, showing the calculated IC50 value. (B) Relative NOX4 mRNA expression in AGS cells treated with icariin at concentrations corresponding to IC25, IC50, and IC75, as determined by qPCR. (C) Representative western blot bands of NOX4 protein expression in AGS cells after icariin treatment. (D) Quantification of relative NOX4 protein expression normalized to GAPDH. (E) Representative fluorescence images showing intracellular ROS levels in AGS cells treated with different concentrations of icariin. (F) Semi‐quantitative analysis of relative intracellular ROS fluorescence intensity in AGS cells after icariin treatment. Data are presented as mean ± SD from three independent experiments (*n* = 3). Statistical analysis was performed using one‐way ANOVA followed by Dunnett's multiple comparisons test. *p < 0.05, **p < 0.01, ***p < 0.001; ns, not significant.

qPCR analysis showed that NOX4 mRNA expression decreased after icariin treatment, and the reduction became more evident with increasing concentrations (Figure 8B). Western blot analysis further indicated that NOX4 protein expression was reduced after icariin treatment, with the most apparent decrease observed in the IC50 group, while the IC75 group remained lower than the NC group overall (Figure 8C,D). In addition, intracellular ROS levels were also reduced after icariin treatment, as shown by fluorescence imaging and semi‐quantitative image analysis (Figure 8E,F). Taken together, these results suggest that icariin may inhibit AGS cell viability and may be associated with reduced NOX4 expression and intracellular ROS levels.

## 4 Discussion

In the present study, we integrated network pharmacology, bulk transcriptomic analysis, single‐cell RNA sequencing, and in vitro experiments to explore the anti‐gastric cancer mechanisms of icariin. NOX4 was identified as a key overlapping target and was associated with poor prognosis in gastric cancer. At the single‐cell level, NOX4 was predominantly expressed in an endothelial subpopulation termed NOX4pos_Endo, which exhibited angiogenesis‐associated transcriptional features. In AGS cells, icariin treatment was associated with reduced NOX4 expression and intracellular ROS levels. These findings suggest that NOX4 may serve as an important node mediating the anti‐tumor effects of icariin in gastric cancer.

NOX4 is a constitutively active member of the NOX family that contributes to intracellular ROS production and redox‐sensitive signaling (Gong et al. 2022). Increasing evidence indicates that oxidative stress has context‐dependent roles in gastric cancer progression, treatment response, and redox‐regulated cell death (Chen et al. 2025; Wang et al. 2024). In our analyses, NOX4 was broadly upregulated across multiple tumor types and was associated with unfavorable survival in three independent gastric cancer cohorts. Its positive association with pathological grade and clinical stage was consistent with previous clinical studies reporting elevated NOX4 expression in gastric cancer tissues and associations with tumor progression, metastatic features, and poor prognosis (Liu et al. 2024; Wang and Gong 2024). These external tissue‐based observations provide additional clinical context for our transcriptomic findings, although they do not replace direct validation in an independent cohort. Mechanistically, NOX4‐derived ROS can influence PI3K/AKT, HIF‐1α, NF‐κB, STAT3, and related pathways involved in proliferation, invasion, angiogenesis, and therapy resistance (Gong et al. 2022). In gastric cancer, NOX4 has also been implicated in anoikis resistance through ROS‐mediated EGFR upregulation (Du et al. 2018) and in the regulation of ferroptosis sensitivity (Wang and Gong 2024). Consistent with these reports, tumors with high NOX4 expression in our study showed enrichment of ECM‐receptor interaction, focal adhesion, and TGF‐β signaling, whereas low‐NOX4 tumors showed stronger associations with fatty acid and pyruvate metabolism. Single‐cell analysis further localized NOX4 predominantly to endothelial cells, in agreement with its established role in vascular biology (Miyano et al. 2020). NOX4pos_Endo cells were characterized by angiogenesis, extracellular matrix remodeling, focal adhesion, integrin signaling, and PI3K‐Akt‐related programs, together with altered oxidative phosphorylation, glycolysis, and glutathione metabolism. CellChat analysis also suggested broader communication between NOX4pos_Endo cells and macrophages, fibroblasts, dendritic cells, and T/NK cells through signaling axes including SPP1, MDK, MIF, and ANGPTL2. These findings are consistent with recent single‐cell studies describing endothelial populations involved in extracellular matrix remodeling and gastric cancer progression (Liu et al. 2025). As well as reports linking NOX4 to VEGF/VEGFR‐2‐dependent endothelial migration (Wang et al. 2024) and TGF‐β1‐mediated endothelial activation (Peshavariya et al. 2014). Importantly, a high NOX4pos_Endo signature score was consistently associated with poorer prognosis across three independent cohorts, supporting the potential clinical relevance of this endothelial state.

Consensus clustering based on the NOX4pos_Endo signature further separated patients into molecular subtypes with distinct biological and clinical features. The subtype with higher NOX4pos_Endo scores showed poorer survival and greater enrichment of EMT, angiogenesis, IL6‐JAK‐STAT3, and inflammatory‐response pathways, whereas the alternative subtype was more strongly associated with DNA repair and metabolic programs. This pattern was reproducible across TCGA‐STAD, GSE84437, and GSE84433. Differences in macrophage, regulatory T‐cell, and mast‐cell infiltration further indicated distinct immune contexts. Consistently, the derived risk groups differed in immune score, stromal score, tumor purity, and the expression of immune‐related molecules including LAG3, PD‐L1 (CD274), and CD276, suggesting that the NOX4pos_Endo‐associated program may reflect broader features of vascular and immune microenvironment remodeling. The five‐gene prognostic model derived from this signature showed moderate predictive performance across cohorts and remained associated with prognosis after multivariate adjustment; however, it should be interpreted as a transcriptional surrogate of the NOX4‐positive endothelial phenotype rather than a direct NOX4‐dependent signature. In vitro, icariin reduced AGS cell viability in a concentration‐dependent manner and was accompanied by decreased NOX4 expression and intracellular ROS levels. Because the observed IC50 of 124.7 μM is substantially higher than systemic concentrations generally achievable in vivo, these findings should be regarded as preliminary evidence under cell‐culture conditions rather than direct evidence of clinical efficacy. Icariin has been reported to exert context‐dependent redox effects in different cellular systems, and therefore the concurrent reductions in NOX4, ROS, and cell viability observed here support an association but do not establish a direct causal mechanism. Molecular docking further suggested a potential interaction between icariin and NOX4 involving Ser576 and Trp377, but this computational result requires biochemical confirmation. Moreover, icariin is a multi‐target compound capable of modulating several cancer‐related pathways (Liu et al. 2023). Although NOX4 was prioritized from 24 overlapping icariin–gastric cancer targets based on its prognostic and single‐cell relevance, it should therefore be considered a candidate regulatory node rather than the sole or confirmed mediator of icariin activity. Further genetic perturbation and direct binding studies will be necessary to define the specific contribution of the icariin–NOX4 axis.

Several limitations should be acknowledged. Experimental validation was restricted to the AGS cell line, while the lack of animal studies and independent clinical tissue validation limits the generalizability and translational relevance of the findings. Intracellular ROS levels were assessed by semi‐quantitative fluorescence microscopy, which may be affected by image acquisition and field selection and therefore requires further validation using more quantitative approaches, such as flow cytometry. In addition, molecular docking and molecular dynamics simulations provide only predictive evidence, and the observed reduction in NOX4 expression and ROS levels does not confirm direct icariin–NOX4 binding. The five‐gene prognostic model reflects the broader transcriptional features associated with NOX4‐positive endothelial cells rather than a direct NOX4‐dependent signature. Further validation in additional gastric cancer cell lines, animal models, clinical specimens, direct binding assays, and quantitative ROS measurements is therefore warranted.

## 5 Conclusion

In conclusion, this study identified NOX4 as a candidate icariin‐associated regulatory node in gastric cancer through integrated bioinformatic, single‐cell, and in vitro analyses. NOX4 was predominantly expressed in an endothelial subpopulation with angiogenesis‐associated transcriptional features, and the corresponding gene signature showed prognostic relevance across multiple cohorts. In AGS cells, icariin treatment was associated with reduced NOX4 expression and intracellular ROS levels. These findings provide a multi‐dimensional characterization of NOX4 in gastric cancer and support further investigation of the potential involvement of NOX4/ROS signaling in the cellular response to icariin.

## Author Contributions

**Na Liang:** conceptualization, methodology, data curation, formal analysis, writing – original draft. **Junyan Li:** conceptualization, methodology, formal analysis, data curation, writing – original draft. **Zuchao Luo:** conceptualization, methodology, software, formal analysis, writing – original draft, visualization. **Zijuan Song:** conceptualization, methodology, validation, formal analysis, writing – original draft. **Liyuan Zhang:** writing – review and editing, validation, investigation. **Chengyi Zou:** writing – review and editing, investigation, validation. **Chunming Li:** conceptualization, writing – review and editing, project administration. **Bochen Pan:** supervision, funding acquisition, project administration, writing – review and editing. **Jiawei Yang:** project administration, writing – review and editing, funding acquisition, supervision, resources.

## Funding

This study was funded by the National Natural Science Foundation of China (32260228, 32460012, and 32560223) and the Guizhou Provincial Science and Technology Department (grant no. QKHJC‐ZK‐2021‐ZD028).

## Ethics Statement

This study was conducted exclusively using publicly available gene expression datasets (TCGA and GEO) and an established human gastric cancer cell line (AGS). All public datasets used in this study were obtained from open‐access repositories and do not contain personally identifiable information. As no human participants, human tissue samples, or animal subjects were involved in this research, ethical approval was not required.

## Conflicts of Interest

The authors declare no conflicts of interest.

## Supporting information


**Figure S1:** Quality control, batch correction, and preliminary clustering of the single‐cell transcriptomic dataset. (A) Distribution of quality‐control metrics across 40 samples (29 tumor samples and 11 normal samples), including nCount_RNA, nFeature_RNA, percent.HB, and percent.mt. (B) Principal component analysis of cells from different samples before batch correction. (C) Low‐dimensional embedding of cells from different samples after Harmony correction. (D) Convergence of the objective function during Harmony iterations. (E) UMAP distribution of cells from different samples after Harmony correction. (F) Unsupervised clustering of the integrated dataset comprising 142,053 cells, identifying 38 cell clusters.
**Figure S2:** Reclustering of epithelial cell subsets and identification of malignant cells based on copy number variation. (A) Reclustering analysis of epithelial cells extracted from the integrated single‐cell datasets. (B) Distribution of CNV scores across epithelial cell subsets. (C) Heatmap of chromosomal copy number variation inferred by inferCNV analysis. (D) Classification of epithelial cells into high‐CNV and low‐CNV groups according to the CNV score threshold. (E) UMAP distribution of high‐CNV and low‐CNV cell populations.
**Figure S3:** Immune microenvironment differences between molecular subtypes defined by the NOX4‐positive endothelial cell signature. (A) Distribution of immune cell infiltration proportions in Cluster 1 and Cluster 2. (B) Comparison of immune cell infiltration between the two subtypes. (C) Differential expression analysis of immune‐related molecules between the 2 subtypes.
**Figure S4:** Consensus clustering and identification of shared NOX4pos_Endo‐related modules across three gastric cancer cohorts. (A, B) Consensus clustering matrix and cumulative distribution function (CDF) curves for the GSE84437 cohort. (C, D) Comparison of NOX4pos_Endo scores and overall survival between the two subtypes in the GSE84437 cohort. (E, F) Consensus clustering matrix and cumulative distribution function (CDF) curves for the GSE84433 cohort. (G, H) Comparison of NOX4pos_Endo scores and overall survival between the 2 subtypes in the GSE84433 cohort. (I–K) WGCNA module‐trait relationship analyses in the TCGA‐STAD, GSE84433, and GSE84437 cohorts, respectively. (L) KEGG enrichment analysis of genes shared by the common cluster‐associated modules across the three cohorts.
**Figure S5:** Association between the five‐gene risk model and the NOX4pos_Endo‐associated transcriptional program. (A–C) Correlations between the risk score and the recalculated NOX4pos_Endo signature score after excluding SPIRE1, PLCH1, KCNS3, SLC27A2, and GRP from the signature gene set in the TCGA‐STAD, GSE84433, and GSE84437 cohorts, respectively. Correlations were assessed using Spearman's correlation analysis.

## Data Availability

The data that support the findings of this study are available from the corresponding author upon reasonable request.
