# Supplementary material for: Integrated Multi‐Omics and Single‐Cell Analyses Implicate NOX4 as an Icariin‐Associated Oxidative Stress Regulatory Node in Gastric Cancer
Source: Food Sci Nutr. 2026 Sep 27;14(10):e72390. doi: 10.1002/fsn3.72390 (PMC13617199; doi:10.1002/fsn3.72390)
Supplement: Supplementary file 1 [file FSN3-14-e72390-s001.docx]

**Figure S1. Quality control, batch correction, and preliminary clustering of the single-cell transcriptomic dataset.** (A) Distribution of quality-control metrics across 40 samples (29 tumor samples and 11 normal samples), including nCount_RNA, nFeature_RNA, percent.HB, and percent.mt. **(B)** Principal component analysis of cells from different samples before batch correction. **(C)** Low-dimensional embedding of cells from different samples after Harmony correction. **(D)** Convergence of the objective function during Harmony iterations. **(E)** UMAP distribution of cells from different samples after Harmony correction. **(F)** Unsupervised clustering of the integrated dataset comprising 142,053 cells, identifying 38 cell clusters.

**Figure S2. Reclustering of epithelial cell subsets and identification of malignant cells based on copy number variation.(A) Reclustering analysis of epithelial cells extracted from the integrated single-cell datasets. (B) Distribution of CNV scores across epithelial cell subsets. (C) Heatmap of chromosomal copy number variation inferred by inferCNV analysis. (D) Classification of epithelial cells into high-CNV and low-CNV groups according to the CNV score threshold. (E) UMAP distribution of high-CNV and low-CNV cell populations.**

**Figure S3. Immune microenvironment differences between molecular subtypes defined by the NOX4-positive endothelial cell signature. (A) Distribution of immune cell infiltration proportions in Cluster 1 and Cluster 2. (B) Comparison of immune cell infiltration between the two subtypes. (C) Differential expression analysis of immune-related molecules between the two subtypes.**

**Figure S4. Consensus clustering and identification of shared NOX4pos_Endo-related modules across three gastric cancer cohorts. (A, B) Consensus clustering matrix and cumulative distribution function (CDF) curves for the GSE84437 cohort. (C, D) Comparison of NOX4pos_Endo scores and overall survival between the two subtypes in the GSE84437 cohort. (E, F) Consensus clustering matrix and cumulative distribution function (CDF) curves for the GSE84433 cohort. (G, H) Comparison of NOX4pos_Endo scores and overall survival between the two subtypes in the GSE84433 cohort. (I-K) WGCNA module-trait relationship analyses in the TCGA-STAD, GSE84433, and GSE84437 cohorts, respectively. (L) KEGG enrichment analysis of genes shared by the common cluster-associated modules across the three cohorts.**

**Figure S5. Association between the five-gene risk model and the NOX4pos_Endo-associated transcriptional program.** **(A–C)** Correlations between the risk score and the recalculated NOX4pos_Endo signature score after excluding SPIRE1, PLCH1, KCNS3, SLC27A2, and GRP from the signature gene set in the TCGA-STAD, GSE84433, and GSE84437 cohorts, respectively. Correlations were assessed using Spearman’s correlation analysis.
